# An Atlas of Bacteriology; Containing 111 Original Photomicrographs with Explanation Text

**Published:** 1899-04

**Authors:** 


AN ATLAS OF BACTERIOLOGY; containing 111 Original photomicrographs with Explanation Text. By CHARLES SLATER, M. A., M. B., M. R. C. S., Eng.; F. C. S., L. R. C. P., London; M. R. C. S., Eng; F. R. A. S.; Formerly Demonstrator of Anatomy, St. George's Hospital Medical School, London. The Scientific Press Ltd., 28 Southampton Street, Strand. 1898. Price, $2.50.

To those students of bacteriology who cannot afford Fraenkel and Pfeiffer's atlas of bacteriology and are not content and satisfied with the reproductions of bacterial life as seen in the ordinary treatises and text-books, this work can be cheerfully recommended. The present work has adopted the idea of the "Atlas" in that it provides a series of photographs of preparations of microörganisms and their cultures, such as are usually met with, and made by the student in an ordinary course of practical bacteriology. It is in no sense a text-book and is not so intended by the authors, but more as a laboratory hand-book to direct the attention of the student to the points which he should observe in his own preparations. The text which is purely explanatory of the plates, emphasises the teachings of the photographs and does not go into the science or art of bacteriology. Although the number of illustrations is of necessity limited, yet it contains 111 original photomicrographs of the commoner forms of microörganisms.

The introduction has been fittingly devoted to a short description of the photographic methods and apparatus employed; the microscope being a Zeiss model 1A with apochromatic objectives, the high magnification, with Powell and Lealand's 1-12 N. A. 1.43. The color screen generally used was a "pot green" glass of medium density and 1-8 inch thick. The negatives were all taken without exception on Edward's isochromatic medium 1-4 plates and developed with hydrokinone and soda. The illustrations are on heavy glazed paper, neatly printed and on the whole it makes a very fine appearance and a desirable work for reference.

